# Evaluation of the Periodontal Status in Acromegalic Patients: A Comparative Study

**DOI:** 10.5402/2012/950486

**Published:** 2012-12-06

**Authors:** Ali N. Harb, Birte Holtfreter, Nele Friedrich, Henri Wallaschofski, Matthias Nauck, Thomas Kocher

**Affiliations:** ^1^Unit of Periodontology, Department of Restorative Dentistry, Periodontology, and Endodontology, Dental School, University Medicine, Ernst Moritz Arndt University of Greifswald, Greifswald 17475, Germany; ^2^Institute of Clinical Chemistry and Laboratory Medicine, University Medicine, Ernst Moritz Arndt University of Greifswald, Greifswald 17475, Germany

## Abstract

*Aim*. The aim was to compare the periodontal status of the acromegalic patients with healthy subjects from a large population-based cohort (Study of Health in Pomerania, SHIP). *Materials and Methods*. We studied 32 acromegalic patients (16 females) and 128 randomly selected SHIP subjects (controls) using a 1 : 4 matching. Serum IGF-I and IGFBP-3 levels were measured using the Immulite 2500 system. Periodontitis was assessed by clinical attachment loss (CAL), probing depth (PD), and number of missing teeth. Linear and logistic regression models were used to assess differences in periodontal variables between acromegalic patients and controls. *Results*. IGF-I levels were comparable in acromegalic patients and controls, whereas IGFBP-3 levels were significantly higher in acromegalic patients (*P* = 0.004). In multivariate modelling, both groups did not differ significantly with respect to mean CAL (*P* = 0.12) and high tooth loss (*P* = 0.36). Mean PD was higher in acromegalic patients by trend (*B* = 0.28 (−0.00; 0.56)). *Conclusion*. In acromegalic patients, periodontal disease severity did not differ from their healthy SHIP controls.

## 1. Introduction

Periodontitis is considered the most common chronic infection in adults. It is an inflammatory disease, which results in alveolar bone loss and degeneration of periodontal tissues [1]. Periodontitis is the leading cause of tooth loss in subjects aged 40+ years [2]. 

Acromegaly is a chronic disease characterized by an acquired progressive somatic deformity, mainly involving the face and extremities [3]. In more than 95% of the cases, acromegaly is caused by a benign pituitary adenoma which develops from the somatotropic cells, leading to hypersecretion of growth hormone (GH) and subsequently to elevated serum levels of insulin-like growth factor hormone-I (IGF-I) [4, 5]. IGF-I is a polypeptide hormone, which is mainly secreted by the liver as a result of stimulation by growth hormones (GH) and mediates most of the endocrine actions of GH [6]. It plays an important anabolic rule in the periodontal ligament and contributes to the regeneration of lost periodontal tissue [7, 8]. Over 90% of IGF-I is bound to IGF binding protein-3 (IGFBP-3) [9]. IGFBP-3 is also found to be increased in patients with acromegaly [3, 10]. Free serum IGF-I is considered the biologically active form of IGF-I [11].

Because of the slow onset and the development of symptoms and because many general practitioners are unfamiliar with acromegaly, there is usually a delay of 7–10 years between beginning of the first symptoms and the diagnosis of the disease at an average age of about 40 years [12]. It was reported that high IGFBP-3 levels are associated with less periodontal disease in the general population [10].

The aim of this study was to compare the periodontal status of the acromegalic patients to the healthy subjects selected from a population-based cohort. Since acromegalic patients probably had been exposed to elevated levels of IGF-I and IGFBP-3 over a decade, we expect them to present less periodontal disease compared to healthy subjects. 

## 2. Material and Methods

### 2.1. Study of Health in Pomerania (SHIP)

The Study of Health in Pomerania (SHIP) is a population-based survey in the north eastern region of Germany. Study details including sampling methods were explained in previous works [13, 14]. In brief, the two-stage cluster design was approved by the World Health Organization Monitoring Trends and Determinants in Cardiovascular Disease (MONICA) project in Augsburg, Germany [15]. Within selected communities, 7008 Caucasian subjects (20–79 years) with German citizenship and main residency in the area were randomly drawn from population registries, stratified by age and sex. Because of several reasons (126 died, 615 moved away, and five had severe medical problems), 746 subjects were excluded, resulting in 6262 citizens being invited to participate. The net random sample included 4,308 subjects (response 68.8%). The study was conducted from October 1997 to May 2001 (SHIP-0). 

The 5-year followup of SHIP-0 was accomplished between 2001 and 2006 (SHIP-1). Healthy controls were selected among SHIP-1 subjects. 

The study protocol was approved a priori by the Ethics Committee of the University of Greifswald. All participants gave informed written consents.

### 2.2. Recruitment of Acromegalic Patients

Forty-one acromegalic patients aged 18–70 years were recruited from the Greifswald University Medical Centre. All of them were treated without growth hormone excess (GH < 1 ng/mL) in oral glucose tolerance test [16]. The same examination protocol as in SHIP was applied. Data collection was performed between September 2006 and March 2007. For 32 subjects complete periodontal examinations and IGF-I measurements were available. 

### 2.3. Oral Health Assessment

Oral examinations were accomplished by trained and calibrated dentists according to the half-mouth method, either on right or left quadrants in alternate subjects. The periodontal status, assessed by CAL and probing depth (PD), was recorded using a periodontal probe (PCP 2; Hu-Friedy, Chicago, IL, USA). Probing was performed at four sites per tooth (mesiobuccal, midbuccal, distobuccal, and midpalatinal/midlingual), excluding the third molars. The number of teeth was counted, excluding the third molars.

Biannual calibration exercises were conducted on subjects not associated with the study yielding intraclass correlations between 0.70–0.89 per examiner and an interrater correlation of 0.90 for attachment level measurements.

### 2.4. Assays of IGF-I and IGFBP-3

IGF-I and IGFBP-3 levels were measured using a chemiluminescent immunometric assay on an Immulite 2500 analyzer (Siemens Immulite 2500, Siemens Healthcare Medical Diagnostics, Bad Nauheim, Germany). Measurements were carried out from April 2008 to May 2008. An aliquot of two levels of the manufacturer's control material (IGF-Control Module, ref. LGCOC, lot 022, Siemens Healthcare Medical Diagnostics, Bad Nauheim, Germany) was included within each series in single determination. During the course of the study the interassay coefficient of variation was 10.8% with a systematic deviation of +6.0% at the 64 ng/mL level for the IGF-I assay and 5.9% with a systematic deviation of +1.1% at the 880 ng/mL level for the IGFBP-3 assay. The IGF-I/IGFBP-3 ratio was calculated.

### 2.5. Assessments of Confounders

Information on sociodemographic characteristics, medical histories, and oral care was retrieved from computer-aided face-to-face interviews. School education was categorised into three levels (<10, 10, and >10 years). Smoking behaviour was assessed with a validated questionnaire and categorised into current, former, and never smokers.

Height, weight, and waist circumference (WC) were measured using calibrated scales. The body mass index was calculated (BMI = body weight (kilograms)/ height^2^ (metres^2^)). Medication with sexual hormones comprised medication with oral contraceptives (ATC code G03A (hormonal contraceptives for systemic use)) and menopausal hormone therapy (Anatomical Therapeutic Chemical (ATC) codes G03C, G03D, or G03F).

Blood pressure was measured three times on the right arm after 5-minute rest in the seated position using a calibrated semiautomatic sphygmomanometer (HEM-705CP; Omron Corporation, Tokyo, Japan) with each reading being followed by a further resting period of three minutes. The average of the last two measurements was used for analysis. Nonfasting serum high-density lipoprotein cholesterol (HDL-C) and serum low-density lipoprotein cholesterol (LDL-C) levels were measured photometrically. Non-fasting serum triglycerides and glucose were determined enzymatically (Hitachi 717, Roche Diagnostics, Mannheim, Germany).

The National Cholesterol Education Program Adult Treatment Panel III (NCEP) criteria for diagnosis of metabolic syndrome [17] were adapted for nonfasting blood samples [18]: WC ≥ 94 cm in males and ≥80 cm in females, serum glucose levels ≥8.0 mmol/L or diabetic medication (Anatomical Therapeutic Chemical (ATC) Code A10), systolic blood pressure ≥130 mmHg and/or diastolic blood pressure ≥85 mmHg or antihypertensive medication (ATC Code C02), serum triglyceride levels ≥2.0 mmol/L or lipid medication (ATC-Code C10AB), and HDL-C <1.03 mmol/L in males and <1.3 mmol/L in females. Individuals who fulfilled at least three or more of these five components were defined to have metabolic syndrome.

### 2.6. Statistical Analyses

A 1 : 4 individual matching by age and sex was used to match 32 acromegalic patients with 128 healthy subjects (referred to as controls) from SHIP-1 (no self-reported cancer, thyroid, liver, or chronic kidney diseases; IGF-I and IGFBP-3 values with the age- and gender-specific reference range [19]). Controls were randomly selected. To enhance efficiency of statistical analyses, matches formed at the design stage were broken and analyses ignored the matching design [20].

Categorical data are presented as percentages; continuous data are presented as median (25%; 75% percentiles). To evaluate differences in the distribution of variables between acromegalic patients and matched controls, Chi-square tests, and Mann-Whitney-*U* tests were evaluated.

Linear and logistic regression models were used to assess differences in periodontal variables between acromegalic patients and healthy SHIP-1 subjects. Linear regression coefficients (*B*) and odds ratios (OR), respectively, with their 95% confidence intervals (CI) were reported. A *P* value <0.05 was considered statistically significant. All analyses were performed using STATA/SE 12.0 [21].

## 3. Results

### 3.1. General Characteristics of Acromegalic Patients

A detailed clinical description of the 32 acromegalic patients is given in Table 1. At least one of the following comorbidities was reported by 62.5% of acromegalic patients: diabetes mellitus, hypertension, chronic kidney disease, chronic thyroid disease, or cancer.

### 3.2. Comparison of Acromegalic Patients with Controls

Compared with controls (Table 2), acromegalic patients had more often diabetes (*P* = 0.04), had higher HDL-C levels (*P* = 0.004), and more often received hormone replacement therapies (*P* < 0.001). Serum IGF-I levels were comparable in both groups, whereas serum IGFBP-3 levels were significantly higher in acromegalic patients compared with controls (*P* = 0.004). Mean CAL, mean PD, and number of missing teeth did not differ significantly between both groups (*P* > 0.05).

According to linear and logistic regression analyses (Table 3), neither mean CAL (*B* = 0.51 (−0.14; 1.17), fully adjusted) nor a high number of missing teeth (OR = 0.59 (0.19; 1.82), fully adjusted) were associated with acromegaly. Mean PD was higher in acromegalic patients by trend (*B* = 0.28 (−0.00; 0.56), *P* = 0.05).

## 4. Discussion

Contrary to our expectations, acromegalic patients had comparable levels of periodontal disease compared to healthy SHIP-1 subjects.

Because only few studies have investigated oral characteristics of acromegalic patients [22, 23], information regarding their periodontal status are rare. A recent Brazilian case-control study evaluated the periodontal status in 32 subjects with untreated congenital GH deficiency (GHD) with low serum IGF-I levels throughout life [24]. GHD subjects had more periodontal disease compared with non-GHD subjects. Another small Brazilian case-control study [25] reported an absence of periodontitis in acromegalic patients. Thus, we expected less (if any) periodontal disease due to higher levels of serum IGF-I in acromegalic patients. In contrast to expectations, periodontal status was comparable between acromegalic patients and controls.

The small sample size in both studies and different diagnostic parameters of periodontal disease may explain the differences in findings between our and the latter studies. Further, interpretation of our results was hampered due to insufficient information about treatment periods in acromegalic patients. Interestingly, treated acromegalic patients had normal levels of serum IGF-I, but significantly higher levels of serum IGFBP-3, compared with controls. However, acromegalic patients also had more comorbidities, which might be expected to aggravate periodontitis. Thus the expected protective effect of higher serum IGF-I levels on periodontitis may have been blurred over the years.

This study has several strengths. First, systemic effects of acromegaly on periodontal disease were evaluated in a relatively large patient sample (*N* = 32). Second, availability of SHIP-1 data enabled selection of a high number of controls applying a 1 : 4 individual matching, which in turn provides higher statistical power. Lastly, different definitions were applied to adequately assess periodontal status in both groups using the same recording protocol.

In conclusion, periodontal disease severity did not differ between acromegalic patients and healthy SHIP-1 controls.

## Figures and Tables

**Table 1:** Detailed clinical description of the 32 acromegalic patients studied.

Patient	Age (years)	Sex	Medication with													
			CB	RA	LP	GB	BAA	AD	PG	HT	PA	TT	SC	IGF-I (ng/mL)	IGFBP-3 (ng/mL)	Comorbidities
I	61	Male			+				+			+	+	133	2980	TH
II	47	Male												204	5870	K
III	61	Male												727	8850	D, H, C
IV	57	Male								+		+	+	138	3960	H
V	48	Male												118	3540	
VI	70	Male							+			+		48.2	1910	
VII	52	Male												75.4	5420	
VIII	48	Male												92.1	2940	H, TH
IX	52	Male							+			+	+	104	3560	TH
X	67	Male		+			+							624	5640	H
XI	53	Male				+		+						249	6180	D, H, C
XII	44	Male				+						+		182	5180	TH, C
XIII	49	Male												314	6260	
XIV	64	Male				+		+						183	4780	
XV	42	Male												148	3680	
XVI	62	Male				+	+							372	6040	H
XVII	41	Female												174	4090	
XVIII	68	Female												191	5450	TH
XIX	61	Female		+			+					+	+	109	6880	D, TH, C
XX	67	Female	+	+	+		+					+		270	7510	D, TH
XXI	63	Female		+	+		+					+		142	5120	H, TH
XXII	36	Female							+					87	3800	
XXIII	47	Female								+	+			126	4300	H, TH
XXIV	23	Female												158	5120	
XXV	69	Female												143	4700	
XXVI	38	Female						+					+	180	4210	
XXVII	63	Female	+			+	+					+	+	199	4980	D, TH
XXVIII	59	Female			+		+	+	+			+	+	86.2	3880	
XXIX	50	Female									+	+		231	5430	C
XXX	59	Female				+								100	4390	D
XXXI	65	Female			+		+	+	+			+	+	41	2220	TH, C
XXXII	70	Female					+				+	+		163	5550	TH

CB: calcium channel blocker; RA: medications affecting the renin-angiotensin system (C09); LP: lipid medication (C10); GB: gall bladder (A05A); BAA: beta-adrenoreceptor antagonists (C07A); AD: androgens (G03B); PG: hormones for pituitary gland (H01A, H01B); HT: hormones for hypothalamus (H01C); PA: psychoanaleptics (N06); TT: thyroid therapy (H03); SC: systemic corticosteroids (H02A).

D: self-reported diabetes mellitus or antidiabetic therapy (ATC code: A10), H: hypertension (defined as diastolic >90 mmH or systolic pressure >140 mmHg or hypertensive medication (ATC code: C02)); K: self-reported chronic kidney disease; TH: self-reported chronic thyroid disease; C: self-reported cancer.

IGF-I: insulin-like growth factor-1.

**Table 2:** Characteristics for acromegaly patients and healthy SHIP-1 subjects.

Variable	Acromegaly patients	Healthy SHIP-1 subjects	P*
*N*	32	128	
Age (years)	58 (48; 64)	58 (48; 64)	0.99
Male gender	50.0%	50.0%	1.00
School education			
<10 years	31.3%	32.0%	
10 years	50.0%	48.4%	
>10 years	18.7%	19.5%	0.99
Smoking status			
Never smoker	40.6%	39.1%	
Former smoker	34.4%	40.6%	
Current smoker	25.0%	20.3%	0.77
Diabetes mellitus**	18.8%	7.0%	0.04
Presence of metabolic syndrome	34.4%	36.7%	0.81
Body Mass Index (kg/m^2^)	28.6 (25.4; 32.7)	28.3 (25.1; 31.1)	0.39
Waist circumference (cm)	92 (86; 104)	95 (86; 102)	0.96
Systolic blood pressure (mmHg)	127 (119; 138)	134 (123; 143)	0.16
Diastolic blood pressure (mmHg)	83 (75; 88)	82 (75; 90)	0.76
Serum glucose (mmol/L)^#^	5.4 (5.0; 6.8)	5.2 (4.8; 5.8)	0.26
Serum triglycerides (mmol/L)^#^	1.5 (1.0; 2.2)	1.6 (1.1; 2.3)	0.49
Serum HDL-C (mmol/L)^#^	1.3 (1.1; 1.6)	1.1 (0.88; 1.4)	0.004
Serum LDL-C (mmol/L)^#^	3.4 (3.0; 4.2)	3.7 (3.0; 4.5)	0.55
Hormone replacement therapy	18.8%	0.0%	<0.001
IGF-I (ng/mL)	153 (107; 2012)	138 (112; 173)	0.19
IGFBP-3 (ng/mL)	4880 (3840; 5595)	4085 (3580; 4625)	0.004
IGF-I/IGFBP-3	0.035 (0.028; 0.041)	0.034 (0.029; 0.042)	0.74
Mean CAL (mm)	2.28 (1.35; 3.67)	2.19 (1.02; 3.39)	0.43
Mean PD (mm)	2.30 (1.92; 2.78)	2.10 (1.87; 2.58)	0.42
Number of missing teeth	7 (2; 11)	6 (2; 12)	0.65
Missing teeth ≥10	28.1%	30.5%	0.80

Data are presented as percentages or median (25% quantile; 75% quantile).

IGF-I: insulin-like growth factor-1; *N*: number; HDL-C: high-density lipoprotein cholesterol; LDL-C: low-density lipoprotein cholesterol; CAL: clinical attachment loss; PD: probing depth.

*Chi-square test (categorical data) or Mann-Whitney- *U* test (continuous data).

**Self-reported physician's diagnosis or antidiabetic treatment (ATC code A10).

^#^Nonfasting blood samples.

**Table 3:** Linear or logistic regression evaluating mean clinical attachment loss, mean probing depth, or high tooth loss (dependent variables) in acromegalic patients compared to SHIP-1 controls.

Model	*B* (95% CI)	*P*
Mean clinical attachment loss		
M0	0.43 (−0.29; 1.15)	0.24
M1	0.44 (−0.18; 1.05)	0.17
M2	0.39 (−0.21; 0.99)	0.20
M3	0.51 (−0.14; 1.17)	0.12

Mean probing depth		
M0	0.23 (−0.04; 0.50)	0.09
M1	0.23 (−0.02; 0.49)	0.07
M2	0.22 (−0.04; 0.48)	0.09
M3	0.28 (−0.00; 0.56)	0.05

Missing teeth ≥10	OR (95% CI)	
M0	0.89 (0.38; 2.11)	0.80
M1	0.88 (0.35; 2.19)	0.78
M2	0.81 (0.31; 2.14)	0.68
M3	0.59 (0.19; 1.82)	0.36

*N*: number.

M0: crude model; M1: adjusted for age (cont.) and gender; M2: adjusted for age (cont.), gender, education, and smoking; M3: adjusted for age (cont.), gender, education, smoking, presence of metabolic syndrome, and medication with sexual hormones.
